# The Centennial of Homœopathy

**Published:** 1896-03

**Authors:** Eugene H. Porter

**Affiliations:** General Secretary A. I. H.; New York City


### THE CENTENNIAL OF HOMŒOPATHY.

New York City, February 29th, 1896.

Editor of The Homœopathic Physician:

The Committee on the Centennial of Homœopathy, Dr. Pemberton Dudley, Chairman, made its final report at the Newport meeting of the American Institute. This report was so thoughtful, and so well considered, that it met the unanimous and instant approval of the Institute, and agreeably to the recommendation contained in the report, I request the aid of your valuable journal in bringing before the homœopathic profession the practical suggestions offered. The report speaks for itself, and I shall therefore quote largely from it; but I may add that the Institute was aroused to an intensely earnest interest, and hoped that the recommendations contained in it would be acted on by the profession. Certainly no more favorable time could be selected to advance the interest of Homœopathy than the present. If in each centre the homœopathic physicians would get together and organize to carry out some of these suggestions of the report great results would follow.

The report says in part: "The American Institute of Homœopathy could hardly feel much enthusiasm in any celebration which had for its object the mere glorification of a man, even though that man were Hahnemann. Still less, probably, would she care to employ such an occasion for the purpose of paying empty compliments to her own members, living or dead. Least of all could this Institute have any patience with the thought of a mere jubilant 'hurrah,' whose influence should end with the last sputter of its expiring fireworks. For any such celebration we have neither the time, the talent, nor the inclination."

In our commemoration of the event of 1796, we should have before us, as its principal object, the promotion of the cause which was then inaugurated. In other words, the celebration should be in strict harmony with the "objects" for which this Institute was organized, as expressed in the opening article of its constitution. In carrying out these objects, we suggest and recommend that the celebration shall be directcd to the following specific purposes, namely:

(*a*) To pay honor to the character, genius, and labor of Hahnemann, and to the work of his discovery.

(*b*) To establish memorials of the man and of his discovery.

(*c*) To re-examine the law of similars in the light of modern knowledge and science.

(*d*) To employ the occasion as a means and opportunity for further extending the knowledge and influence of Homœopathy, and for imparting a new impetus to its development.

The central thought of the celebration should be the discovery promulgated in 1796—the law of similars. Public and professional attention should be drawn as strongly as possible to this particular subject as the distinctive and essential "truth" of Homœopathy, while other truths taught by Hahnemann, and held by his followers, should, for the time being, occupy a secondary place. This sharp distinction should be made for the purpose of forcing public and professional recognition of the real and essential question at issue between the two methods of medical practice.

In the view of your committee, the celebration should not be restricted to the national society, but in certain ways should be co-extensive with our country, and its influence maintained throughout the centennial year.

We recommend that, so far as the Institute is directly concerned, the arrangements and details of the celebration should be in charge of a committee consisting of the Executive Committee of the years 1895 and 1896 acting conjointly.

We also recommend that the duties of the said Joint Committee should include the following :

(*a*) The Committee should prepare a circular, giving an outline of the proposed celebration, including all the recommendations adopted by the Institute in relation thereto, and send copies thereof, not later than December 15th, 1895, to all the homœopathic journals published in the United States, requesting its publication in the first issue of 1896, together with editorial comment upon the subject, and also requesting each journal to publish, during the year, such further favorable comment as its Editor might deem expedient.

(*b*) The Committee should recommend in said circular that each State and local society provide a celebration of its own, of such character as to draw public attention to the Centennial of Homœopathy and the important results of Hahnemann's Law of Cure.

(*c*) Also, that the friends of each homœopathic hospital in the United States should, during the year, endow at least one bed in perpetuity, to be so designated and inscribed as to constitute a permanent memorial of the Centennial, and of the event which it celebrates.

(*d*) Also, that each city and large town, not already provided with a homœopathic hospital, should, during the year, inaugurate a movement to secure such an institution.

In addition, the Institute, in accordance with the suggestion of the report, celebrate the Centennial of Homœopathy by a public meeting, when the address, " The Hahnemann Oration," shall be delivered by the President.

Three Centennial addresses on the Law of Similars will also be delivered as follows:

1. " The Rational Basis of the Law of Similars."

2. " The Experimental Demonstration of the Law of Similars."

3. " The Clinical Superiority and Efficacy of the Law of Similars."

It will be seen that this celebration will lend increased interest to the Detroit meeting. By interesting local newspapers in the matter, and making public the needs of the Hahnemann Monument Committee, much-needed aid may be had. This report, so timely and so suggestive, will, I trust, be acted upon by your readers and receive your cordial support.

Fraternally yours,

EUGENE H. PORTER,
*General Secretary A. I. H.*

